# Odorant Responses and Courtship Behaviors Influenced by at4 Neurons in Drosophila

**DOI:** 10.1371/journal.pone.0162761

**Published:** 2016-09-12

**Authors:** Svetlana Pitts, Elizabeth Pelser, Julian Meeks, Dean Smith

**Affiliations:** 1 Department of Neuroscience, University of Texas Southwestern Medical Center, 5323 Harry Hines Blvd., Dallas, TX 75390–9111, United States of America; 2 Department of Pharmacology, University of Texas Southwestern Medical Center, 5323 Harry Hines Blvd., Dallas, TX 75390–9111, United States of America; USDA-ARS Beltsville Agricultural Research Center, UNITED STATES

## Abstract

In insects, pheromones function as triggers to elicit complex behavior programs, such as courtship and mating behavior. In most species, the neurons tuned to pheromones are localized in a specific subset of olfactory sensilla located on the antenna called trichoid sensilla. In Drosophila there are two classes of trichoid sensilla, at1 sensilla that contain the dendrites of a single neuron that is specifically tuned to the male-specific pheromone 11-*cis* vaccenyl acetate (cVA), and at4 sensilla that contain three neurons with relatively poorly defined chemical specificity and function. Using a combination of odorant receptor mutant analysis, single sensillum electrophysiology and optogenetics, we have examined the chemical tuning and behavioral consequences of the three at4 olfactory neuron classes. Our results indicate that one class, Or65abc neurons, are unresponsive to cVA pheromone, and function to inhibit courtship behaviors in response to an unknown ligand, while the other two neuron classes, Or88a and Or47b neurons, are sensitive to a diverse array of fly and non-fly odors, and activation of these neurons has little direct impact on courtship behaviors.

## Introduction

Social behaviors in insects are genetically-encoded, stereotypic responses that are often triggered by pheromones. Non-volatile pheromones are detected by specialized gustatory sensilla, often located on the legs, and detect sex and species-specific cuticle components through members of the pickpocket family [1–4]. Volatile pheromones in most insects are detected by olfactory neurons located on the antenna, in a class of specialized hair-like structures called trichoid sensilla [5]. Understanding the scope and function of volatile pheromones in insects may lead to future targets to manipulate insect behaviors.

In *Drosophila melanogaster*, there are two classes of trichoid sensilla, at1 and at4. at1 and at4 sensilla can be unambiguously identified based on the number of neurons they house and the odorant receptors expressed by these neurons [6]. at1 sensilla contain the dendrite of a single neuron that expresses Or67d odorant receptors. These neurons are exquisitely tuned to the male-specific pheromone, 11-cis-vaccenyl acetate (cVA), forming a dedicated ‘labeled line’ to recognize the presence of cVA pheromone [7–9]. cVA is detected by both sexes but is only produced by males [10, 11], and its detection occurs on a scale of millimeters to centimeters among individuals, mediating recognition of conspecifics as male or female [12]. Indeed, males lacking the Or67d receptors are insensitive to cVA and display increased male-to-male courtship while females show reduced mating receptivity [9, 13]. In addition to sexually dimorphic courtship behavior, exposure of wild type animals to cVA mediates male-male aggression and social aggregation at food sources [9, 14–16].

The function of the three neurons clustered in at4 sensilla are much less well understood. These sensilla house the dendrites of three olfactory receptor neurons, each expressing different receptors. These at4 neurons express either Or47b, Or88a or three genetically linked receptors, Or65a, Or65b and Or65c (Or65abc) [6]. The receptors expressed in at4 neurons have been implicated in the detection of fly-derived odors [17–22], suggesting they may function as dedicated circuits for specific fly-derived pheromones. However, the chemical specificity and roles proposed for the at4 neurons varies among the studies, often resulting in contradictory conclusions. Here, we have evaluated the chemical sensitivity of the at4 neurons and have used mutants and optogenetic stimulation of these circuits to assess their potential roles in courtship behaviors.

## Materials and Methods

### Drosophila stocks

An isogenized strain of *w*^*1118*^ was used as a wild type control for SSR and courtship behavior experiments. ∆Or65abc was produced by crossing flies carrying the FRT containing transposon insertions XP(d06290) at 65A11 with flies carrying the RB(e00271) insertion at 65B1 in the presence of the FLP recombinase gene induced by heat shock. Recombinant progeny were identified by a double dose of the *w+* minigene present in the recombinant chromosome and confirmed by loss of *Or65a*, *b* and *c* genes by PCR and the presence of a 2.4 kb PCR fragment spanning the breakpoint. *Or47b*^-^ stocks were previously reported by Wang et al. [21] and were provided to us by Leslie Vosshall. The *Or88a* mutant (*Or88a*^-^) was made using CRISPR/Cas9 [23]. We targeted the first exon of *Or88a* at the genomic sequence GACCTGATGTGCACCacTTGCGG with the PAM site underlined. A two-nucleotide deletion (lower case letters) was recovered, producing a frame-shift and premature termination in the predicted receptor. *Or47b-Gal4* and *Or65a-Gal4* lines were obtained from the Bloomington Stock Center (stocks 9983 and 9993 respectively). *UAS-Or83c* line was described in [24]. The *Or67d*^*Gal4*^ knock-in was described by Kurtovic [9]. The red-shifted channelrhodopsin *UAS-ReaChR* lines were reported by Inagaki et al. [25] and obtained from Bloomington (stocks 53749 and 53741).

### Odorant preparation

Compounds used in single sensillum recordings were of the highest purity available (Sigma-Aldrich and Pherobank BV). In order to prepare olfactory stimulus, 30 μl of diluted or undiluted odorant were placed on a small piece of filter paper, which was inserted into a 5.75 inch Pasteur pipette. Farnesol and cVA were used undiluted to maximize the response. Odorants used to obtain the tuning curves were diluted with paraffin oil and used at 10% dilution on filter paper. One hundred fifty *w*^*1118*^ male or virgin female flies in a Pasteur pipette were used as a source of fly odorants. For the odorant list, see S1 and S2 Tables.

### Single sensillum electrophysiology

**Fig 1:**
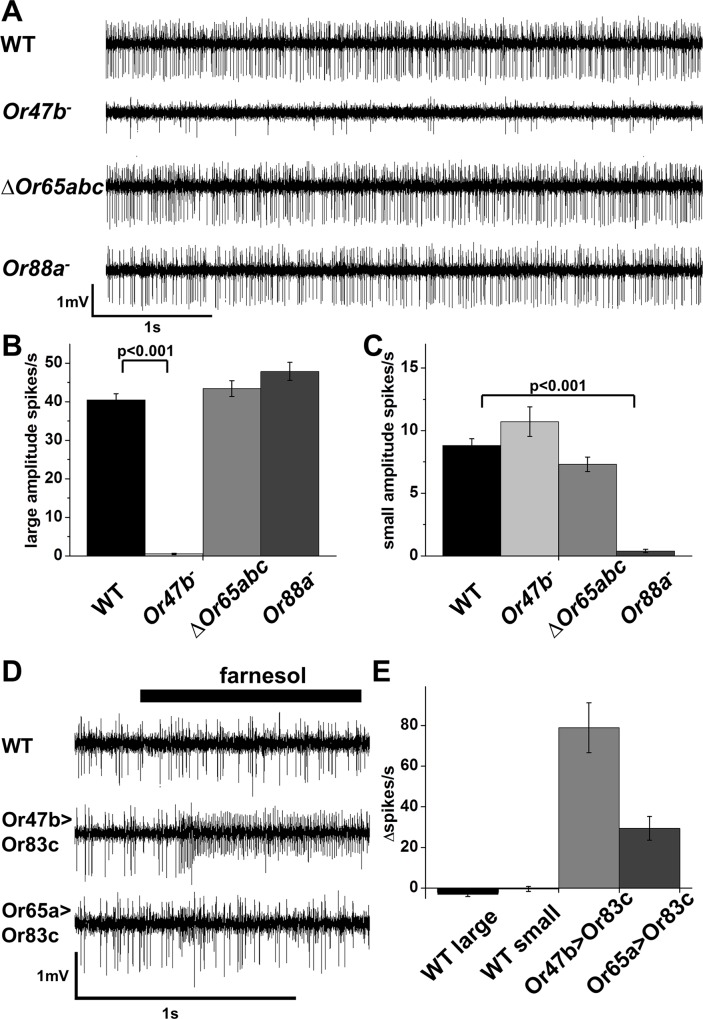
Correlating spike amplitudes with neurons expressing specific receptors. A. Representative traces from at4 sensillum neurons from wild type (WT), *Or47b* mutant (*Or47b*^*-*^) *Or65abc* knock-out (∆*Or65abc*^*-*^) and *Or88a* mutant (*Or88a*^*-*^) flies. B. Average spontaneous activity of neurons producing large amplitude spikes in at4 of WT, *Or47b*^*-*^, *∆Or65abc* and *Or88a*^*-*^ flies (n = 26). *Or47b*^*-*^ is significantly different from control, p<0.001 C. Average spontaneous activity of neurons producing small amplitude spikes in at4 WT, *Or47b*^*-*^, ∆*Or65abc* and *Or88a*^-^ flies (n = 26). *Or88a*^*-*^ is significantly different from control, p<0.001. D. Representative traces showing response of at4 neurons to farnesol in WT, *Or47b-Gal4;UAS-Or83c* (*Or47b>Or83c*) and *Or65a-Gal4;UAS-Or83c* (*Or65a>Or83c*) flies. E. Changes in number of large (WT, *Or47b>Or83c*) and small (WT, *Or65a>Or83c*) amplitude spikes in response to farnesol, n = 6 for WT and *Or47b>Or83c*, n = 9 for *Or65a>Or83c*. Error bars indicate SEM.

**Fig 2:**
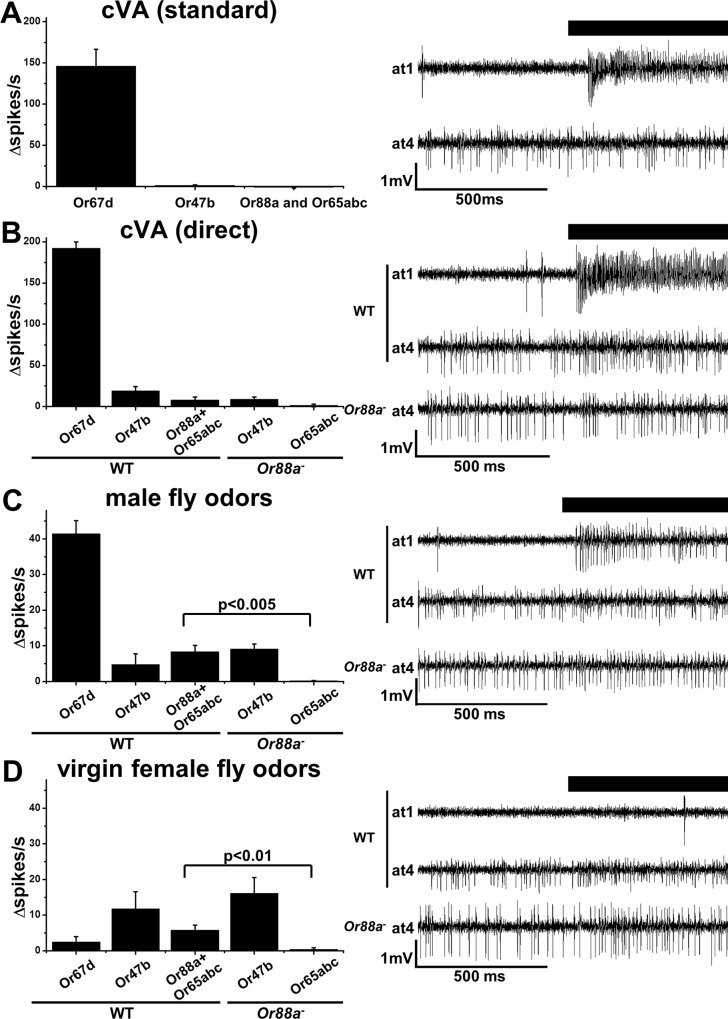
Response profiles of Or47b, Or88a and Or65abc to fly odors. A. Responses of at1 and at4 neurons from WT and *Or88a*^-^ mutants to cVA applied in standard conditions (n = 4). Representative traces are shown on the right. B. Responses of WT at1 (n = 4) and at4 (n = 5), and *Or88a*^*-*^ at4 sensilla (n = 10) neurons to undiluted cVA vapors. Representative traces are shown on the right. C. Responses of WT at1 and at4 neurons and *Or88a*^*-*^ at4 neurons to odors emitted by 150 living male flies (n = 6). Changes in frequency of small amplitude spikes are significantly different between *Or88a*^*-*^ and controls, p<0.005. Representative traces are shown on the right. D. Responses of WT at1 (n = 6), at4 (n = 6) and *Or88a*^*-*^ at4 neurons (n = 5) to odors emitted by 150 living virgin female flies. Changes in frequency of small amplitude spikes are significantly different between *Or88a*^*-*^ and controls, p<0.01. Error bars indicate SEM. Representative traces are shown on the right.

Single sensillum recordings were performed as described in [12] except time of application was extended to a 1s air pulse and flies of both sexes were used at ages 1–4 days, except for flies used for optogenetic experiments that were used at 5–6 days old to allow for retinal ingestion. Flies were housed in fresh vials containing standard yeast molasses food in small groups prior to SSR recordings. Flies used for optogenetic experiments were housed in the dark on Nutri-Fly media containing retinal (see Optogenetic and Behavioral Experiments below for details). Spike waveforms from the recordings were sorted based on amplitude and shape using custom software written in MATLAB [26, 27]. An example of the sorting program is shown in S1 Fig. In brief, putative spikes were identified based on user-defined amplitude thresholds, which were typically two-fold greater than the root mean square noise in the absence of stimulation. Principal component analysis was performed for on all putative spike waveforms (3 ms around each spike). Spikes were then initially sorted using k-means clustering and were then manually merged into large-amplitude and small-amplitude populations. The independence of the large- and small-amplitude spike populations was further confirmed by evaluating refractory periods in graphs of temporal autocorrelation and cross-correlation. ‘Direct’ application of cVA was performed by applying a 1 second air pulse through the Pasteur pipet containing cVA and this air was applied directly to the fly. Odors from living flies were also applied directly in order to maximize the response. All recorded responses to each stimulant were obtained from separate sensilla, with a maximum of three sensilla recorded from any single fly. Sensilla were identified based on anatomical location, sensillum morphology, spontaneous activity, and odorant sensitivity. For Fig 1, values were compared using one-way ANOVA. The Dunnett test was used to correct for multiple comparisons. The analysis was performed using GraphPad Prism. For Fig 2, the two-tailed Student T-test was used to compare *Or88a*^-^ mutants with controls. Analysis was performed in Origin 8.5. A 633nm laser (Uniphase) was used to stimulate the *Or>ReaChR* flies (S2 Fig).

### Optogenetic and behavioral experiments

All flies used in mating behavior assays were collected as virgins and kept at 25°C in constant dark until eclosion in standard vials. Controls were *w;+;UASReaChR*. Males were housed individually, and females were housed in small groups in fresh vials. A stock solution of 40 mM all-trans-retinal (Sigma) in DMSO was stored in -20°C. Male flies for optogenetic experiments were placed on fly food (Nutri-Fly) containing 1% of the retinal stock solution for 5–6 days from eclosion until the assay was performed. Females were raised on standard food.

Single naive virgin male and female flies were placed in courtship chambers (1.5-cm-diameter polystyrene wells containing 1.5 ml of 1% agarose in water covered with paper) and covered with glass coverslip. The 30-minute video was recorded using a Canon powershot 4000 HD camera. Light source used for optogenetic experiments was adapted from [25]. High-power red (627 nm) Rebel LEDs (LUXEON) was placed at a 12 cm distance from the courtship chamber and turned on as soon as flies were introduced to the chamber. Courtship index (CI = time spent in courtship/total time) for all experiments was measured by a co-worker blinded to genotype. CI was calculated as a portion of time male fly is engaged in any step of the courtship (tracing/chasing, orienting, wing vibration/courtship song, abdominal curling, and copulation) in the first 10 minutes of the assay. Mating latency was calculated as percentage of males that successfully copulated in a group of 10 for every minute of the assay. Two-way ANOVA was used to compare CI and mating latency in presence and absence of red light. Tukey test was used to correct for multiple comparison. Analysis was performed in GraphPad Prism.

## Results

### Or47b neurons produce large amplitude action potentials, while Or65abc and Or88a neurons spike with small amplitudes but distinct spontaneous frequencies

Single sensillum recordings from unstimulated at4 sensilla are complex due the presence of spontaneous activity from all three neurons within the sensillum. Two distinct classes of spikes are present based on spike amplitude and shape (Fig 1). However, we do not know what neurons expressing specific receptors correspond to what spikes in the recordings.

Odorant receptors in Drosophila are heteromultimers composed of a common subunit, Orco (Or83b), and one of 60 tuning receptor subunits that provides chemical specificity to the complex [28, 29]. Olfactory neurons lacking expression of tuning receptor subunits have drastically reduced spontaneous activity rates and loss of odorant sensitivity [9, 29, 30]. Therefore, to correlate neurons expressing specific receptors with specific spike amplitudes in at4 recordings we compared the spontaneous activity produced by neurons in wild type at4 sensilla with those of mutants lacking specific receptors expressed by at4 neurons. We examined the spontaneous activity of at4 neurons in flies defective for Or47b receptor expression. Single sensillum recordings from mutants lacking Or47b (*Or47b*^*-*^) are defective for large-amplitude spontaneous spikes in at4 sensilla recordings (40± 2 spikes/s for wild type and 0.5 ±0.2 spikes /s), indicating these large spikes arise from Or47b-expressing neurons (Fig 1A and 1B). The at4 recordings from CRISPR mutants lacking Or88a (*Or88a*^*-*^) have no reduction in the number of large-amplitude action potentials, but a highly significant reduction in the frequency of small amplitude spikes (8.8 ± 0.5 spikes/s in wild type and 0.4 ±0.1 spikes/s in *Or88a*^*-*^ mutants, Fig 1A and 1C). This suggests Or88a neurons are responsible for the majority of the small amplitude action potentials observed in at4 recordings (Fig 1A and 1C). Mutants lacking all three Or65a, b and c receptors were produced by inducing recombination between two FRT-containing transposons that flank the receptors producing a small deletion encompassing all three receptor genes (see Materials and Methods). at4 recordings from mutants homozygous for the *Or65abc* receptor deletion have no change in the number of large amplitude spikes, and a small reduction in the average number of small amplitude spikes (Fig 1A–1C). To confirm the spike amplitudes of the three at4 neurons we mis-expressed the farnesol receptor Or83c [24] in each neuron class and assayed the responses to farnesol. Wild type at4 neurons do not respond to farnesol (Fig 1D and 1E) Mis-expression of Or83c in the Or47b neurons results in an increase in the number of large amplitude spikes upon farnesol exposure (Fig 1D and 1E). Farnesol exposure of Or65abc neurons expressing Or83c increases the number of small amplitude spikes (Fig 1D and 1E). Therefore we conclude that Or65abc neurons produce small amplitude spikes and that this neuron has a low endogenous spontaneous activity rate, while Or88a neurons also have small amplitude spikes but have a relatively high rate of spontaneous activity.

### Or47b and Or88a are broadly tuned, while Or65abc neurons are narrowly tuned

Having correlated electrophysiological signatures to neurons expressing specific receptors, we next explored the ligand specificity of these receptors in their natural neuronal environment. We examined the responses of the at4 neurons to fly volatiles as well as a panel of odorants. First, we examined the sensitivity of these neurons to fly odors. cVA has been reported to directly activate Or65a receptors mis-expressed in basiconic neurons lacking tuning receptors [20]. Furthermore, cVA has been reported to modulate mating behavior through Or65abc neurons [17, 18, 31]. Therefore, we examined the responses of at4 neurons to cVA. cVA pheromone potently activates the at1 Or67d neurons [15] (Fig 2A). Surprisingly, even at the highest cVA concentrations applied using our standard delivery system (see Materials and Methods) we observed no stimulation or inhibition on any of the three at4 neurons (Fig 2A). To test the possibility that one or more of the at4 neurons are cVA detectors, but are less sensitive to cVA than at1 neurons, we applied undiluted cVA vapors directly to the at4 sensilla and recorded the responses. Under these conditions, we did observe a slight increase in the frequency of both large and small amplitude spikes (Fig 2B). To identify whether the Or65abc, the Or88a neuron or both respond to concentrated cVA, we tested the cVA response in *Or88a*^-^ receptor mutant flies. In these mutants, the only small amplitude spikes in at4 sensilla are generated from Or65abc neurons. We observed no increase in small action potentials to undiluted cVA in these flies, revealing the Or88a neurons are responsible for these cVA responses. The increase in action potentials elicited from Or47b and Or88a neurons by cVA are only observed at concentrations that are higher than would be expected under normal conditions, and even at these levels Or65abc neurons are unresponsive to cVA.

Both male and female fly odors have been reported to active at4 neurons [20, 22]. Indeed, wild type at4 neurons responded to air passed over male or virgin female flies (Fig 2, see Materials and Methods). There is a significant increase in spike frequencies from both large and small amplitude neurons in response to application of both male and female odors The responses to male odors are 7.4 ±3 ∆spikes/s for large and 8.3 ±1.9 ∆spikes/s for small amplitude spikes. The responses to female odors are 11.7 ± 4.8 ∆spikes/s for large and 5.8 ± 1.4 ∆spikes/s for small amplitude spikes. (Fig 2C and 2D). We repeated the experiment on *Or88a*^-^ flies to establish the contribution of the Or65abc-expressing neurons. In the absence of Or88a, we observed no change in the frequency of small amplitude spikes in response to the male or female odors (Fig 2C and 2D). Therefore, Or88a neurons and Or47b neurons, but not Or65abc neurons respond to odors emitted from both sexes. However, while female odors produce more robust activation of Or47b neurons compared to male odors, there appears to be no qualitative sex differences in at4 neuron responses. It should be noted that the responses of the at4 neurons to these stimuli are much weaker than those elicited from the Or67d neurons to male odors, consistent with previous studies [20].

### Or47b neurons respond to a large number of odorants

**Fig 3:**
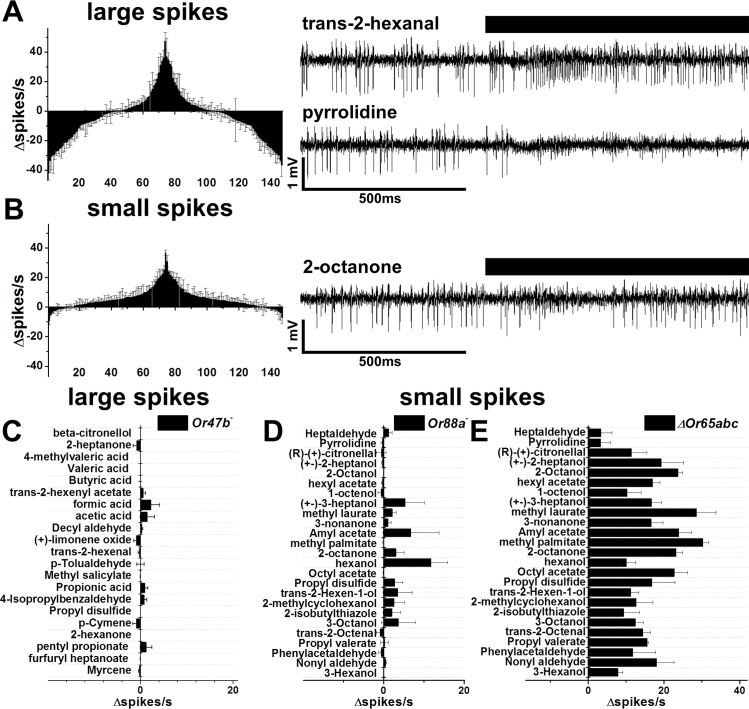
Response profiles of Or47b, Or88a and Or65abc to non-fly odors. A. Tuning curve for Or47b receptor neuron. Changes in number of large amplitude spikes in response to each of 147 odorants are shown (n ≥ 3 for each odorant). Representative traces evoked by the strongest activating and inhibiting odorant are shown on the right. B. Changes in amount of small amplitude spikes in response to each of 147 odorants are shown (n ≥ 3). Representative traces evoked by the strongest activating and inhibiting odorants are shown on the right. C. Changes in amount of large amplitude spikes in response to Or47b activators in *Or47b*^*-*^ mutant flies (n ≥ 3 for each odorant). Odors are listed in the same order as presented in the peak in A. D. Changes in amount of small amplitude spikes in response to Or88a and Or65abc activators in *Or88a*^*-*^ mutant flies (n ≥ 2 for each odorant). Odors are listed in the same order as presented in the peak in B. E. Changes in amount of small amplitude spikes in response to Or88a and Or65abc activators in ∆*Or65abc* flies (n ≥ 2 for each odorant). Error bars indicate SEM.

Recent studies concluded that Or47b neurons are dedicated circuits for the fly cuticle component methyl laurate, and that this odorant influences courtship behavior [22]. However, the responses of Or47b neurons to general odorants were not tested. We screened an odorant library containing 147 different compounds to identify potential activators and inhibitors of at4 neurons, including the large-spiking Or47b neurons (Fig 3). A number of odorants were identified that induced an increase in the number of large or small amplitude spikes (≥10 ∆spikes/s) that were then tested on *Or47b*^-^, ∆Or65abc and *Or88a*^-^ flies to establish the odorant sensitivity curves for individual at4 neurons (Fig 3C–3E). From a panel of 147 odorants we identified twenty-one odorants that stimulated Or47b neurons ≥10 spikes/s. Interestingly, methyl laurate, while an activator, was not among the most potent activators. Surprisingly, over forty ligands inhibited action potentials in this neuron. This suggests that Or47b neuron could potentially transmit biological information not only by being activated, but also by being inhibited [32].

Twenty-five odors were identified that produced small amplitude spikes ≥ 10 ∆spikes/sec. To resolve which neuron responded to each odorant, we tested these compounds in Or88a^-^ and ∆Or65abc mutants. All twenty-five odorants activate Or88a neurons, and one odorant was identified that activated both Or88a and Or65abc-expressing neurons (Fig 3B and 3D and 3E). Both Or47b and Or88a neuron classes responded to odorants with diverse chemical structures, including the methylated cuticle lipids recently described [22]. By contrast, Or65abc neurons appear to be narrowly tuned, responding to only 1-hexanol, but not to the other odorants in our panel, including cVA. Application of even concentrated cVA reveals that Or65abc neurons are completely insensitive to cVA pheromone (Fig 2). Hexanol is known to be an odorant that activates many classes of olfactory neurons in the fly [32], and thus is not a specific activator of Or65abc neurons. These results indicate that Or47b neurons and Or88a neurons are sensitive to a diverse array of ligands, including both fly and non-fly derived compounds.

### Activation of Or65abc neuron inhibits courtship and delays mating

**Fig 4:**
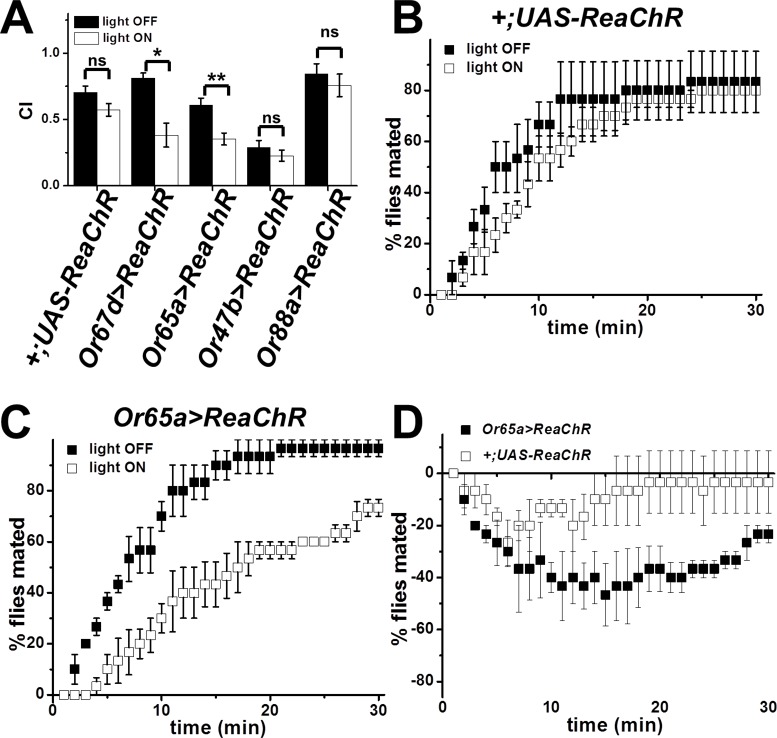
Activation of Or65abc neuron decreases courtship and delays mating. A. Courtship indexes of control *(+;UAS-ReaChR*), *UAS-ReaChR;Or67d*^*Gal4*^ (*Or67d>ReaChR*) and *Or65a-Gal4;UAS-ReaChR* (*Or65a>ReaChR*), males paired with WT virgin female with and without red light. n = 31, for *+;UAS-ReaChR*, n = 9 for *Or67d>ReaChR* and n = 29 for *Or65a>ReaChR*. *p<0.05; **p<0.01 B. Latency to mate of control (*+;UAS-ReaChR*) males paired with WT virgin females with and without red light. Mating latency is represented as the percentage of mated flies out of the group of ten pairings, n = 3. C. Latency to mate of *Or65a-Gal4;UAS-ReaChR* (*Or65a>ReaChR*) males paired with WT virgin females with and without red light. Mating latency is represented as the percentage of mated flies out of the group of ten pairings, n = 3. D. Light-dependent delay in mating of *Or65a>ReaChR* compared to *+;UAS-ReaChR* flies. The graph was obtained by subtracting results observed in the absence of light from the results observed under red light stimulation. *Or65a>ReaChR* are significantly different from *+;UAS-ReaChR*, p<0.0001, 2-way ANOVA. Error bars indicate SEM.

To explore the potential roles for the at4 neurons in courtship, we tested the effect of specifically activating individual neuron classes using red light and the red-shifted channelrhodopsin, ReaChR [25, 33]. Red light activates each of the neuron classes in which we expressed ReaChR (S2l Fig). We measured the courtship index of control, *Or67d>ReaChR*, *Or88>ReaChR*, *Or47b>ReaChR* and *Or65a>ReaChR*–expressing flies with and without red light stimulation. We first examined the behavioral responses of flies expressing ReaChR in the cVA-sensing Or67d neurons. Previous studies have demonstrated that activation of these neurons in males with cVA inhibits courtship behavior [13]. Therefore, we predicted that stimulation of these neurons should inhibit courtship behavior in males. Indeed, activation of Or67d neurons with red light results in a significant reduction in courtship index (CI) compared to light-off conditions in genetically identical animals (Fig 4A). We next examined the effect of stimulating the Or47b neurons. We observed no statistically significant differences in courtship index between red light stimulated and unstimulated controls in *Or47b>ReaChR* flies (Fig 4A). We also observed no difference in courtship index in flies expressing ReaChR in Or88a neurons with or without red light. However, we did see a striking reduction in courtship index in the flies expressing ReaChR in the Or65abc neurons in the presence of red light (Fig 4A).

To further examine the courtship phenotype induced by activation of Or65abc, we monitored whether red light stimulation leads to alterations in overall mating success. This assay measures both mating latency and the ability to complete the courtship program. We measured the cumulative mating success for *Or65a>ReaChR* male flies with and without red light. Control animals show similar mating kinetics with and without light (Fig 4B). Consistent with the reduction in courtship index we observed, red light activation of ReaChR in Or65abc neurons significantly reduced mating success (Fig 4C and 4D). These data reveal that activation of Or65abc inhibits courtship, and results in delayed mating.

## Discussion

Previous studies examining the odorant specificity of the receptors expressed in at4 sensilla neurons and the function of the circuits activated by these neurons have produced different theories about chemical specificity and biological roles. Activation of Or65abc neurons by cVA has been reported to affect learning, aggression, aggregation and courtship behaviors [17, 18, 31]. Chronic exposure to cVA was found to reduce male-male aggression and this behavior was attributed to Or65a-expressing neurons [31]. Lebreton et al. showed that recently mated females are no longer attracted to cVA, and silencing the Or65abc neurons prevent this post-mating change, speculating that prolonged exposure of cVA activates Or65abc neurons [18]. Ronderos et al. found excess cVA in the courtship chamber inhibited male-female courtship in an Or67d-dependent manner [13], Ejima et al. concluded that Or65abc neurons and not Or67d neurons are responsible for the decrease in male-to-female courtship in the presence of cVA [17]. Here, we show that endogenous Or65abc neurons inhibit mating behavior when stimulated, but that cVA is not able to stimulate them. This implicates some other, as yet unknown ligand that normally inhibits mating that is detected through these neurons. This ligand is not likely to be a fly-derived odor, as neither male nor female fly odors are capable of activating Or65abc neurons.

One of the first published reports examining the chemical specificity of at4 neurons reported the ability to activate at4 neurons with hexane extracts from both male and female cuticles, but these workers were not able to stimulate the at4 neurons with volatile fly odors [20]. This work also reported that cVA was capable of activating at4 neurons, although a higher concentration was required than for activating at1 neurons. However, they were not able to distinguish which neurons expressing specific receptors in the at4 sensilla were stimulated. Misexpression of Or65a, one of the three receptor genes expressed in Or65abc neurons [6], in basiconic neurons lacking endogenous receptors was found to weakly sensitize these neurons to cVA, suggesting the at4 neurons responding to cVA may be the Or65abc neurons, but this stimulation required direct contact of cVA liquid with the sensillum [20].

In our studies, high concentrations of cVA are able to elicit weak responses from at4 neurons. We observed increases in activity from both large and small spiking neurons. However, in the absence of Or88a with intact Or65abc neurons, there is no increase in small spikes, revealing Or65abc neurons lack cVA sensitivity, and the weak cVA response in small spiking neurons is derived from Or88a. Therefore, while none of the at4 neurons respond strongly to cVA, the Or65abc neurons are cVA insensitive. Indeed, Or65abc neurons did not respond to any of the ligands in our library with the exception of hexanol. This odorant is known to activate a large number of Drosophila olfactory neurons [32]. We conclude that the Or65abc neurons are narrowly tuned, do not respond to cVA, and may be activated by some unknown compound.

Interestingly, despite the fact that the Or65abc neuron does not respond to fly odorants or most of the odorants in our library as we have applied them, this neuron has a strong negative effect on courtship behavior. One explanation is that Or65abc detects an important negative environmental factor, that acts to over-ride courtship behavior when conditions are not appropriate. One intriguing possibility is that Or65abc responds to some life-threatening danger such as the presence of a predator, when mating behavior would reduce fitness. While we can define the role of Or65abc in inhibiting mating behavior through optogenetics, we do not know the biologically relevant ligand that induces this behavior in nature. Considering the striking effect of activation of this neuron on the behavior, it would be very interesting to identify its ligand.

In recent work, GC-MS was used to identify cuticle components and revealed methyl laurate, methyl palmitate and methyl myristate are minor cuticle compounds that active at4 neurons [22]. These compounds are present in both male and female cuticle, and are detected by both sexes, and therefore are not mating cues. These compounds are present in many Drosophila species, indicating they are unlikely to be species-specific cues. The large-spiking Or47b neurons are selectively activated by methyl laurate, but not methyl myristate or methyl palmitate, and activation of these neurons is associated with increased mating success [22]. These studies concluded that Or47b represents a labeled line for methyl laurate, which acts as a positive cue to promote mating. However, they did not examine the sensitivity of Or47b neurons to general odorants.

We found a similar sensitivity profile to methylated lipids in Or47b and Or88a neurons, but find that neither class is dedicated to the detection of these compounds, but instead are activated by a wide array of odorants. Indeed, several of our odorants are better activators of Or47b neurons than methyl laurate at a similar concentration. Indeed, methyl laurate failed to activate Or47b neurons above our cut-off of 10 spikes per second, and would be considered a weak activator in our screen. Since both fly and non-fly odorants affect the activity of Or47b neurons, Or47b is not a labeled line for methyl laurate as is the case for Or67d neurons that detect cVA pheromone exclusively [8, 9, 15]. The broad tuning of Or47b neurons is supported by recent work from Munch and Galizia who examined fluorescence activation of glomeruli innervated by Or47b neurons in flies exposed to a library of 100 odorants [34]. These workers found a number of weakly activating and inhibitory olfactory ligands [34]. We and Munch note that many odorants inhibit Or47b or its downstream circuits, indicating that suppression of this neuron may also have biological relevance [32, 35]. Dweck et al. observed a reduction in mating success in males lacking Or47b receptors and expressed trpA1 in the Or47b neurons and observed increased mating success at higher temperatures when these flies competed for mates with wild type controls [22]. We did not observe a significant positive or negative effect on courtship index upon activating the Or47b neurons optogenetically, which provides a much better temporal control over neuronal activity. However, since the competition assays used in Dweck et al. are different from the courtship assays we performed, we can not rule out a small effect of Or47b neurons on mating behavior [22]. However, it should be noted that since Or47b neurons are responsive to fly and non-fly derived odors it more likely that Or47b-activated circuits have an indirect effect on reproductive behavior.

Or88a neurons are activated by a large number of ligands, including the methylated compounds identified by Dweck et al.[22]. We observe broad tuning of Or88a neurons, and the sensitivity to these compounds is lost in Or88a mutants. Similar to what we observed with optogenetic activation of Or47b neurons, optogenetic activation of Or88a neurons also did not affect courtship index. This finding is consistent with the conclusions of Dweck et al. who concluded that Or88a mediates attraction behavior and not social behavior in response to the presence of these methylated lipids [22].

In summary, we have established that Or47b and Or88a neurons in at4 sensilla are broadly tuned receptor neurons that modulate their activity to both fly and non-fly odorants, and conclude that optogenetic activation of these neurons has little effect on courtship behavior. Furthermore, we show that endogenous Or65abc neurons are insensitive to cVA, are narrowly tuned, but activation of these neurons results in inhibition of courtship behavior. We were unable to identify ecologically relevant ligands for this neuron in our odorant library, but identifying a specific activator could provide insight into Drosophila ecology and potentially have applications for controlling mating behavior in pest species.

## Supporting Information

S1 Fig Large/small amplitude spike sorting. A. SSR showing spontaneous spiking in a wild-type at4 sensillum. Blue/red vertical ticks: times of sorted large/small amplitude spikes. B. Expanded view of the region indicated by the gray box in Panel A. C. Overlaid waveforms of all large and small amplitude spikes from the SSR shown in Panel A (10 s total). D. Projections of the sorted waveforms along the first and second principal components (PC1 and PC2, respectively). Each colored dot indicates the projection of an individual spike (same SSR shown in Panels A-C). E. Histogram of d’ values for 529 wild-type SSRs. d’ values were calculated from linear discriminant analysis (LDA) projections. Red dotted lines indicate the d’ values associated with the statistical thresholds of p = 0.05 (100% of recordings) and p = 0.01 (96.4% of recordings; z-score approximations).
(PDF)

S2 Fig Transgenic flies expressing ReaChR are activated by red light. Representative traces show red light activation of *Or47b>ReaChR*, *Or65a>ReaChR* and *Or88a>ReaChR* flies. The raster plot under *Or47b>ReaChR* trace corresponds with positions of large amplitude spikes, and the raster plots under *Or65a>ReaChR* and *Or88a>ReaChR* traces indicate positions of small amplitude spikes.
(PDF)

S1 Table Odorant-dependent changes in the amount of large amplitude spikes. (PDF)

S2 Table Odorant-dependent changes in the amount of small amplitude spikes. (PDF)
