# Correction: Odorant Responses and Courtship Behaviors Influenced by at4 Neurons in Drosophila

**DOI:** 10.1371/journal.pone.0190805

**Published:** 2018-01-02

**Authors:** Svetlana Pitts, Elizabeth Pelser, Julian Meeks, Dean Smith

The following information is missing from the funding section: This study was supported by NIH grant 5T32GM008203 to EP.
