# Supplementary material for: Odorant Responses and Courtship Behaviors Influenced by at4 Neurons in Drosophila
Source: PLoS One. 2016 Sep 12;11(9):e0162761. doi: 10.1371/journal.pone.0162761 (PMC5019410; doi:10.1371/journal.pone.0162761)
Supplement: S1 Fig — A. SSR showing spontaneous spiking in a wild-type at4 sensillum. Blue/red vertical ticks: times of sorted large/small amplitude spikes. B. Expanded view of the region indicated by the gray box in Panel A. C. Overlaid waveforms of all large and small amplitude spikes from the SSR shown in Panel A (10 s total). D. Projections of the sorted waveforms along the first and second principal components (PC1 and PC2, respectively). Each colored dot indicates the projection of an individual spike (same SSR shown in Panels A-C). E. Histogram of d’ values for 529 wild-type SSRs. d’ values were calculated from linear discriminant analysis (LDA) projections. Red dotted lines indicate the d’ values associated with the statistical thresholds of p = 0.05 (100% of recordings) and p = 0.01 (96.4% of recordings; z-score approximations). (PDF) [file pone.0162761.s001.pdf]

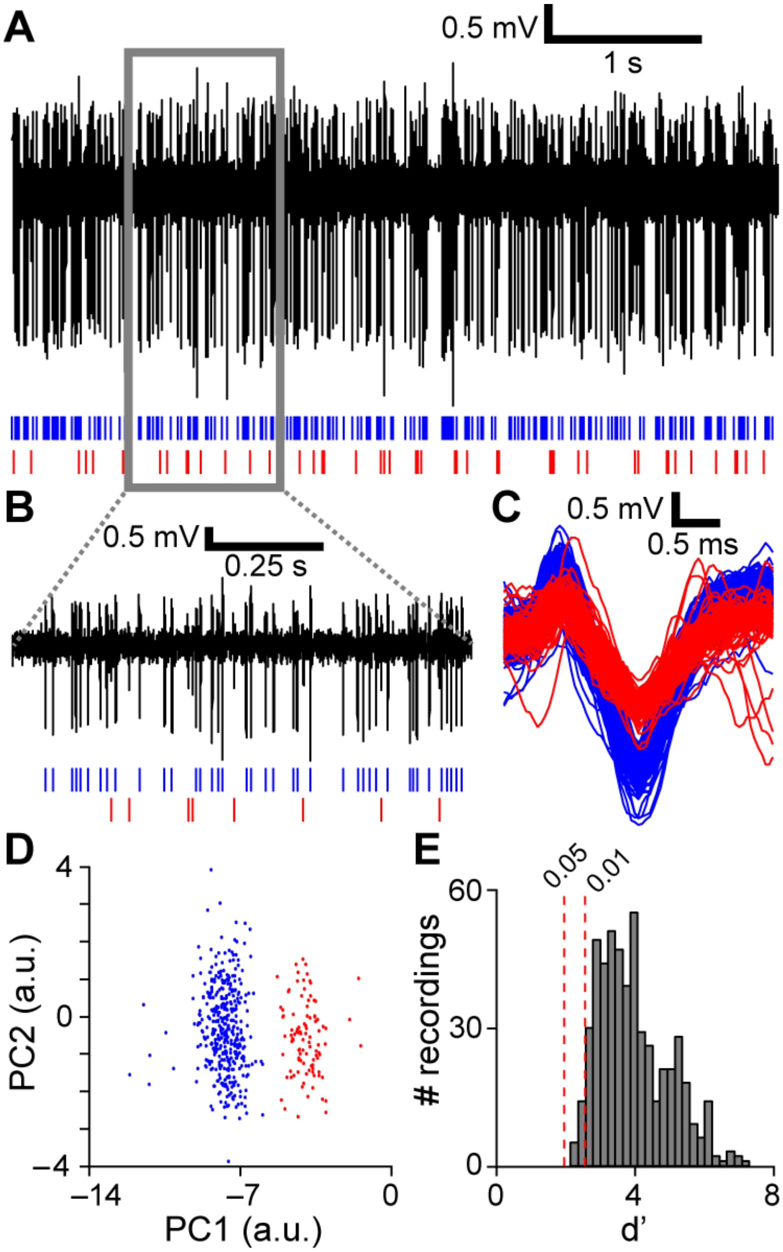

Supplemental Fig 1) Large/small amplitude spike sorting. (**A**) SSR showing spontaneous spiking in a wild-type at4 sensillum. Blue/red vertical ticks: times of sorted large/small amplitude spikes. (**B**) Expanded view of the region indicated by the gray box in Panel A. (**C**) Overlaid waveforms of all large and small amplitude spikes from the SSR shown in Panel A (10 s total). (**D**) Projections of the sorted waveforms along the first and second principal components (PC1 and PC2, respectively). Each colored dot indicates the projection of an individual spike (same SSR shown in Panels A-C). (**E**) Histogram of d' values for 529 wild-type SSRs. d' values were calculated from linear discriminant analysis (LDA) projections. Red dotted lines indicate the d' values associated with the statistical thresholds of $p = 0.05$ (100% of recordings) and $p = 0.01$ (96.4% of recordings; *z*-score approximations).
