# Supplementary material for: Odorant Responses and Courtship Behaviors Influenced by at4 Neurons in Drosophila
Source: PLoS One. 2016 Sep 12;11(9):e0162761. doi: 10.1371/journal.pone.0162761 (PMC5019410; doi:10.1371/journal.pone.0162761)
Supplement: S2 Fig — Representative traces show red light activation of Or47b>ReaChR, Or65a>ReaChR and Or88a>ReaChR flies. The raster plot under Or47b>ReaChR trace corresponds with positions of large amplitude spikes, and the raster plots under Or65a>ReaChR and Or88a>ReaChR traces indicate positions of small amplitude spikes. (PDF) [file pone.0162761.s002.pdf]

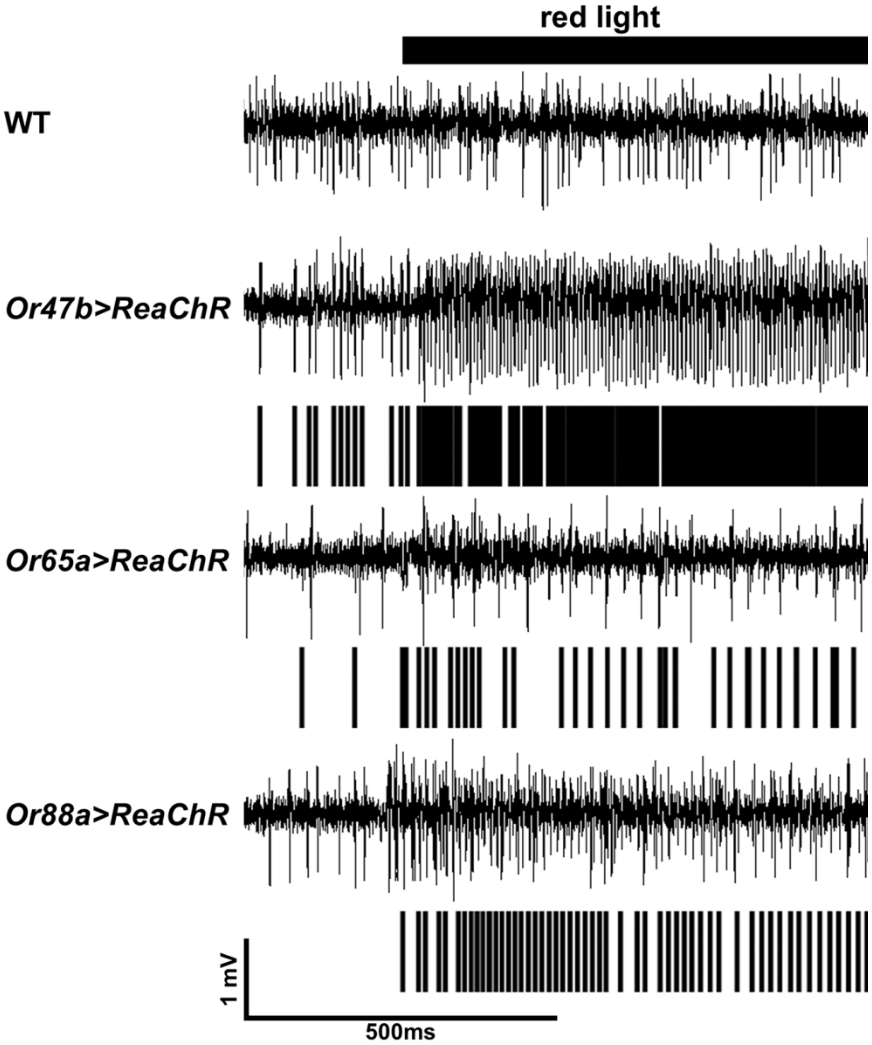

Supplementary Fig 2. Transgenic flies expressing ReaChR are activated by red light. Representative traces show red light activation of *Or47b>ReaChR*, *Or65a>ReaChR* and *Or88a>ReaChR* flies. The raster plot under *Or47b>ReaChR* trace corresponds with positions of large amplitude spikes, and the raster plots under *Or65a>ReaChR* and *Or88a>ReaChR* traces indicate positions of small amplitude spikes.
