# Supplementary material for: Odorant Responses and Courtship Behaviors Influenced by at4 Neurons in Drosophila
Source: PLoS One. 2016 Sep 12;11(9):e0162761. doi: 10.1371/journal.pone.0162761 (PMC5019410; doi:10.1371/journal.pone.0162761)
Supplement: S1 Table — (PDF) [file pone.0162761.s003.pdf]

Supplementary Table 1. Odorant-dependent changes in the amount of large amplitude spikes ranked from best activator to best inhibitor.

odorant	response, Δspikes/s
trans-2-hexenal	47±6
p-tolualdehyde	39±4
(+)-limonene oxide	37±10
methyl salicylate	34±3
acetic acid	32±2
decyl aldehyde	31±5
formic acid	30±3
propionic acid	29±6
trans-2-hexenyl acetate	22±3
propyl disulfide	22±3
4-Isopropylbenzaldehyde	21±9
butyric acid	20±6
p-cymene	19±3
2-hexanone	18±3
valeric acid	16±3
pentyl propionate	15±10
2-heptanone	14±10
furfuryl heptanoate	13±5
4-methylvaleric acid	13±6
beta-citronellol	11±2
myrcene	10±10
4-methylcyclohexanol	10±6
trans-anethole	9.2±0.5
l-bornyl acetate	9±6
(±)-menthol	7.8±0.9
(R)-(+)-citronellal	7±1
methyl laurate	7±3
(R)-(+)-limonene	6±5
(R)-(-)-carvone	6±5
isoamyl alcohol	6±3
2-methylquinoxaline	6±3
methyl anthranilate	5±2
2-octenoic acid	5±2
citronellyl acetate	4±3
geranyl acetate	4±3
phenethyl alcohol	4±4
furfuryl octanoate	4±2
isovaleric acid	4±2
2-acetylthiazole	4±2

linalool	3±1
hexanoic acid	3±3
acetophenone	3±4
nonanoic acid	3±3
4-allyl-1,2-dimethyl benzene	2±2
toluene	2±3
phenylacetaldehyde	2±7
2-isobutylthiazole	2±6
pentane	1±2
dimethoxymethane	1±2
hexane	1±3
octanoic acid	1±2
propanol	1±1
6-heptenoic acid	0.8±0.1
(-)-carveol	1±3
linalyl acetate	1±4
octyl acetate	1±3
2-propanol	0±2
cresol	0±5
1,1-dimethoxyethane	0±5
valeraldehyde	-0.1±0.8
1-propanol	0±2
2,3-dimethylpyrazine	0±3
acetone	0±1
valencene	0±1
methyl sulfoxide	-1±1
4,5-dimethylthiazole	-1±4
ethyl formate	-1±2
isoamyl acetate	-1±2
cineole	-1±8
2-octanone	-1±6
2-methylcyclohexanol	-1±2
methyl isobutyrate	-1±3
α-ionone	-2±2
2-pentanone	-2±3
amyl acetate	-2±6
citral	-2±3
benzyl benzoate	-2±1
2,3-butanedione	-2±2
2-butanol	-3±3
methyl palmitate	-3±5
geraniol	-3.8±0.2
(+-)-2-butanol	-4±3

catnip	-4±2
2-methoxypyrazine	-4±2
2-butanone	-5±0.4
octyl aldehyde	-5±2
2-methylpyrazine	-6±2
2-decanone	-6±14
cyclohexanone	-6±2
propyl propionate	-6±2
ethyl 2-methylbutanoate	-7±2
farnesol	-6.8±0.8
benzyl alcohol	-7±3
propyl valerate	-7±6
2-isobutyl-3-methoxypyrazine	-7±3
ethyl acetate	-8±2
methyl alcohol	-8±2
14-diaminobutane	-8±1
benzaldehyde	-8±7
2-methoxy-3-methylpyrazine	-8±5
2-methyl-1-propanol	-8±9
butyraldehyde	-8±3
nonyl aldehyde	-9±4
γ-valerolactone	-9.2±0.4
3-nonanone	-9±3
butylamine	-9±3
7-oxabicyclo[2.2.1]-heptaine	-10±3
3-heptanone	-10±3
γ-valerolactone	-11.5±0.4
2,6-dimethylpyrazine	-12±5
3-octanone	-14±4
propyl acetate	-14±2
3-hexanol	-15±4
hexanal	-15±4
butyl acetate	-16±5
hexanol	-17±4
4-methylthiazole	-17±2
1-octenol	-18±8
ethylpyrazine	-19±5
3-methyl-1-butanol	-19±8
cis-2-hexen-1-ol	-19±5
trans-2-octenal	-20±4
2-pentanol	-21±2
1-octen-3-ol	-21±7
thiazole	-21±4

1-nonanol	-21±3
2-octanol	-21±6
butyl butyrate	-24±3
salicylaldehyde	-24±5
ethyl butyrate	-24±4
(+-)-2-heptanol	-25±7
2,4,5-trimethylthiazole	-25±5
ethyl propionate	-26±5
(+-)-3-heptanol	-26±3
pentyl acetate	-27±2
eugenol	-27±3
1-pentanol	-27±3
heptaldehyde	-28±7
3-octanol	-29±4
hexyl acetate	-29±6
1-butanol	-30±10
hexylamine	-30±2
benzylamine	-31±9
cis-2-hexen-1-ol	-34±1
2-hexanol	-34±8
trans-2-hexen-1-o	-37±8
pyrrolidine	-42±5
