# Supplementary material for: Odorant Responses and Courtship Behaviors Influenced by at4 Neurons in Drosophila
Source: PLoS One. 2016 Sep 12;11(9):e0162761. doi: 10.1371/journal.pone.0162761 (PMC5019410; doi:10.1371/journal.pone.0162761)
Supplement: S2 Table — (PDF) [file pone.0162761.s004.pdf]

Supplementary Table 2. Odorant-dependent changes in the frequency of small amplitude spikes ranked from best activator to best inhibitor.

odorant	response, ∆spikes/s
2-octanone	37±2
methyl palmitate	31±4
hexanol	25±3
amyl acetate	24±0.7
octyl acetate	22±3
3-nonanone	22±2
propyl disulfide	21±3
methyl laurate	21±3
trans-2-hexen-1-ol	20±6
(+-)-3-heptanol	19±4
2-methylcyclohexanol	19±5
1-octenol	19±2
2-isobutylthiazole	18±3
hexyl acetate	17±5
3-octanol	15±3
2-octanol	15±5
trans-2-octenal	14±3
(+-)-2-heptanol	14±6
propyl valerate	12.9±0.6
(R)-(+)-citronellal	13±3
phenylacetaldehyde	12±2
pyrrolidine	12±6
nonyl aldehyde	11±3
heptaldehyde	10±2
3-hexanol	10±4
1-pentanol	10±1
(R)-(-)-carvone	9.9±0.3
2-hexanone	10±6
p-cymene	9±5
4-methylvaleric acid	9.3
pentyl acetate	9±5.6
3-methyl-1-butano	8.4±0.7
octyl aldehyde	8±5
4-methylcyclohexanol	8±4
1-nonanol	8±5
myrcene	8±6
furfuryl heptanoate	8±5
linalool	8±2
hexylamine	7±4

L-bornyl acetate	7±1
cis-2-hexen-1-ol	7±3
trans-2-hexenyl acetate	7±3
trans-anethole	7±3
2-methoxypyrazine	7±4
butyl butyrate	7±3
isoamyl alcohol	7±3
isovaleric acid	7±1
cyclohexanone	6.6±0.3
2-hexanol	7±3
ethylpyrazine	6±6
beta-citronellol	6±4
2-decanone	6±4
toluene	6±2
2-heptanone	6±3
hexanoic acid	6±7
2-methyl-1-propanol	5.6±0.9
2-acetylthiazole	5±5
isoamyl acetate	5±4
2-butanol	5±3
cineole	5±5
butyl acetate	5±4
4-isopropylbenzaldehyde	5±1
butyric acid	5±3
1-propanol	5±1
2-pentanol	5±3
decyl aldehyde	5±3
7-oxabicyclo[2.2.1]-heptaine	4.8±0.8
acetophenone	5±3
ethyl propionate	5±3
3-octanone	5±5
ethyl 2-methylbutanoate	5±2
phenethyl alcohol	4±3
citronellyl acetate	4±4
butylamine	4.3±0.3
(+-)-2-butanol	4±2
3-heptanone	4±4
(R)-(+)-limonene	4±3
p-tolualdehyde	4±4
acetic acid	4±1
2-methylpyrazine	4±2
valeric acid	4±2
(±)-menthol	4±3

propyl propionate	4±1
valencene	4±2
octanoic acid	4±3
benzyl alcohol	4±2
salicylaldehyde	4±1
trans-2-hexenal	3±2
methyl anthranilate	3.4±0.7
linalyl acetate	3±3
citral	3±2
methyl sulfoxide	3±2
pentyl propionate	3±4
methyl alcohol	3±1
γ-valerolactone	3±1
(-)-carveol	3±1
2-propanol	2.8±0.3
ethyl butyrate	3±6
2-methylquinoxaline	3±3
1-octen-3-ol	3±3
benzaldehyde	2±3
eugenol	2±3
valeraldehyde	2±1
hexane	2±2
6-heptenoic acid	2±2
2-pentanone	2±2
propanol	2±4
hexanal	2±1
α-ionone	2±4
propionic acid	2±2
nonanoic acid	2±1
14-diaminobutane	1±3
geranyl acetate	1±3
methyl salicylate	1±4
4,5-imethylthiazole	1±2
butyraldehyde	1±3
benzylamine	0.3±0.1
benzyl benzoate	0±1
dimethoxymethane	0±1
furfuryl octanoate	0±2
cresol	0±2
geraniol	0±6
methyl isobutyrate	-0.1±0.8
formic acid	0±2
1-butanol	0±2

propyl acetate	0±3
2,3-butanedione	0±2
pentane	-0.4±0.8
4-methylthiazole	-0.8±0.3
2,6-dimethylpyrazine	-0.8±0.1
acetone	-1.2±0.9
2-octenoic acid	-1.2±0.8
catnip	-1±1
1,1-dimethoxyethane	-1±1
2,3-dimethylpyrazine	-2±3
ethyl formate	-2±1
4-allyl-1,2-dimethyl benzene	-2±4
2-butanone	-2±1
ethyl acetate	-2.4±0.3
farnesol	-2±2
(+)-limonene oxide	-3±2
2-isobutyl-3-methoxypyrazine	-3±5
thiazole	-3.6±0.2
ethyl butyrate	-4±5
2-methoxy-3-methylpyrazine	-5.2±0.8
2,4,5-trimethylthiazole	-7±4
3-heptanone	-10±2
